# In silico investigation of brain tissue responses associated with mild blast traumatic brain injury

**DOI:** 10.3389/fbioe.2026.1880112

**Published:** 2026-08-20

**Authors:** Tarun Sachdeva, C. Jayarami Reddy, S. G. Ganpule

**Affiliations:** 1 Department of Mechanical Engineering, Indian Institute of Technology Roorkee, Roorkee, India; 2 Defence Metallurgical Research Laboratory, DRDO, Hyderabad, India; 3 Department of Mechanical Engineering, Indian Institute of Technology Bombay, Mumbai, India

**Keywords:** critical brain regions, finite element analysis, mild blast traumatic brain injury, primary blast, regional brain biomechanical responses

## Abstract

Blast induced traumatic brain injury (bTBI) is a significant concern in the era of tactical warfare. The biomechanical response of the human head to blast loading is actively sought. While blast wave propagation through brain tissue is reasonably well understood, the direct relationship between external blast parameters and brain biomechanical responses remains unclear. In this study, a 3D computational human model was used to investigate these relationships under free-field blast conditions relevant to mild bTBI, with peak incident pressure (IOP:70–145 kPa) and positive phase duration (PPD:2–8 ms). The blast wave head interactions were modeled using the conventional weapon (ConWep) technique for a duration of 80 ms. Front and side orientations were considered. The interaction of the blast wave with the human head induced a biomechanical cascade within the brain, which can be broadly categorized into wave propagation and inertial effects. Each of these effects had a peculiar timescale and induced distinct biomechanical signatures. The relationship between external blast parameters and brain biomechanical parameters indicated that brain pressure and the maximum principal strain rate (MPSR) were correlated with peak IOP, whereas equivalent stress and the maximum principal strain (MPS) were correlated with blast impulse. These correlations were specifically governed by the wave propagation and inertial effects, respectively. An increase in PPD had a more deleterious effect than an increase in peak IOP. Brain biomechanical responses produced higher values in the side orientation than in the front orientation, particularly affecting midbrain regions, including the corpus callosum (CC). Brain region specific analysis revealed that pressure was concentrated in the cerebrum (C), whereas equivalent stress and MPS were concentrated in CC. Comparison with blunt TBI suggested similarities in the spatiotemporal evolution of equivalent stress and MPS, but differences in pressure evolution. Overall, these results provide important new insights into the brain’s response to the blast and enhance the overall mechanistic understanding of bTBI.

## 1 Introduction

Blast induced traumatic brain injury (bTBI) continues to be a major threat during asymmetric warfare (Tanielian, 2008; Jones et al., 2007). Mild bTBI was identified as a signature wound during conflicts in Iraq and Afghanistan (Owens et al., 2008; Belmont et al., 2010). More than 2 decades have passed since the early research publications of mild bTBI (Cernak et al., 2001a; Cernak et al., 2001b; Trudeau et al., 1998; Sachdeva and Ganpule, 2024). Since then, considerable progress has been made in understanding mild bTBI through experiments, computational modeling, imaging, and clinical studies. Broadly, four different mechanisms: wave transmission (Ganpule et al., 2013; Nyein et al., 2010; Sundaramurthy et al., 2012; Taylor and Ford, 2009), head acceleration (Aravind et al., 2020a; Goldstein et al., 2012; Risling et al., 2011; Gullotti et al., 2014), skull flexure (Bolander et al., 2011; Garimella et al., 2018; Moss et al., 2009), and thoracic (Cernak et al., 2001a; Moss et al., 2009; Koliatsos et al., 2011) have been proposed for the mild bTBI. Out of these, wave transmission and head acceleration were considered significant in humans. Skull flexure and thoracic mechanisms were especially peculiar in early bTBI investigations in rodents, especially due to the lack of robust test protocols (Cernak, 2010; Long et al., 2009; Säljö et al., 2008; Tompkins et al., 2013; Xu et al., 2016).

The pathology and behavioral changes in mild bTBI have been mainly investigated using rodent models. Blood-brain barrier (BBB) permeability, oxidative stress, diffuse axonal injury (DAI), and multifocal axonal damage are commonly observed pathological features (Abdul-Muneer et al., 2013; Garman et al., 2011; Hue et al., 2013; Kabu et al., 2015; Readnower et al., 2010; Cheng et al., 2010; Kuehn et al., 2011; Ahlers et al., 2012). Anxiety (Sajja et al., 2015; Cernak et al., 2011; Awwad et al., 2015; Kamnaksh et al., 2011; Russell et al., 2018; Oleksiak et al., 2012; Swan et al., 2017; Yurgil et al., 2016; Galazyuk and Hébert, 2015), memory (Goldstein et al., 2012; Long et al., 2009; Rubovitch et al., 2011; Song et al., 2018; Stemper et al., 2016; Beamer et al., 2016), motor deficits (Oleksiak et al., 2012; Swan et al., 2017; Yurgil et al., 2016; Luo et al., 2014; Mao et al., 2012), and fear conditioning (McDannald, 2010; Park et al., 2013; Pickens et al., 2010) are consistently reported behavioral deficits. Numerous clinical investigations are available in combat veterans. These studies have reported symptoms such as headache, sleep disorder, poor motor skills, cognitive dysfunction, depression, and anxiety (Borinuoluwa and Ahmed, 2023; Dismuke-Greer et al., 2020; McGlinchey et al., 2017; Siedhoff et al., 2022; Walker et al., 2018; Belanger et al., 2010; Elbogen et al., 2014). Neuroimaging studies among military personnel have predominantly reported loss of white matter integrity (Hayes et al., 2015; Mac Donald et al., 2013; Miller et al., 2016; Riedy et al., 2016; Robinson et al., 2015; Edlow et al., 2022; Mac Donald et al., 2011), diffuse axonal injury (DAI) (Mac Donald et al., 2011; Ryu et al., 2014; Vakhtin et al., 2013; Shively et al., 2016), alterations in the thalamic network (Elbogen et al., 2014), and cortical thickness (Stone et al., 2020; Wang et al., 2020; Hellewell et al., 2023; Clark et al., 2018; Eierud et al., 2019; Lindemer et al., 2013; Martindale et al., 2021; Tate et al., 2014; Vartanian et al., 2021). We also note that investigations using rodents outweigh those in humans, especially given the difficulty and ethical constraints of obtaining data in humans.

Despite the aforementioned progress, the relationship between external blast stimulus, typically in the form of a Friedlander wave, and the corresponding biomechanical response of the human brain is not fully understood. Investigations using three-dimensional (3D) computational models of the human head offer an alternative for studying the effects of the blast wave and the resulting biomechanical cascade, provided the models give reasonable predictions. Such models can offer significant insights into biomechanics and injury quantification. Sutar and Ganpule (2024) extensively validated the 3D human head model across a range of blast scenarios and studied the mechanics of blast wave head interaction. However, the relationship between external blast parameters and brain specific biomechanical parameters was not explicitly investigated. This understanding is important for determining whether a direct correlation exists between blast parameters and specific brain biomechanical parameters. Further, this understanding is critical for quantifying whole brain and region specific biomechanical responses with their associated vulnerabilities as a function of external blast parameters.

Towards this end, we investigated the relationship between external blast parameters associated with mild bTBI and corresponding brain biomechanical parameters. The entire blast spectrum (IOP: 70–145 kPa, with PPD: 2–8 ms) was simulated. From these simulations, we derive key observations regarding the relationship between external blast stimulus and internal biomechanical response of the brain. We also quantified values of various brain biomechanical parameters for the whole brain and a few regions of interest. The manuscript is organized as follows. In Section 2, we describe a 3D full body finite element human model and simulated loading conditions, along with the boundary conditions; we further explain the data analysis and its reduction. In Section 3, the results are presented. In Section 4, results are discussed in order to understand the relationship between the blast parameters and the brain biomechanical parameters and their regional impacts. In Section 5, the limitations of this study are mentioned. Finally, in Section 6, based on the results and discussion, the conclusions of this work are laid down.

## 2 Methods

### 2.1 Finite element human model

**FIGURE 1:**
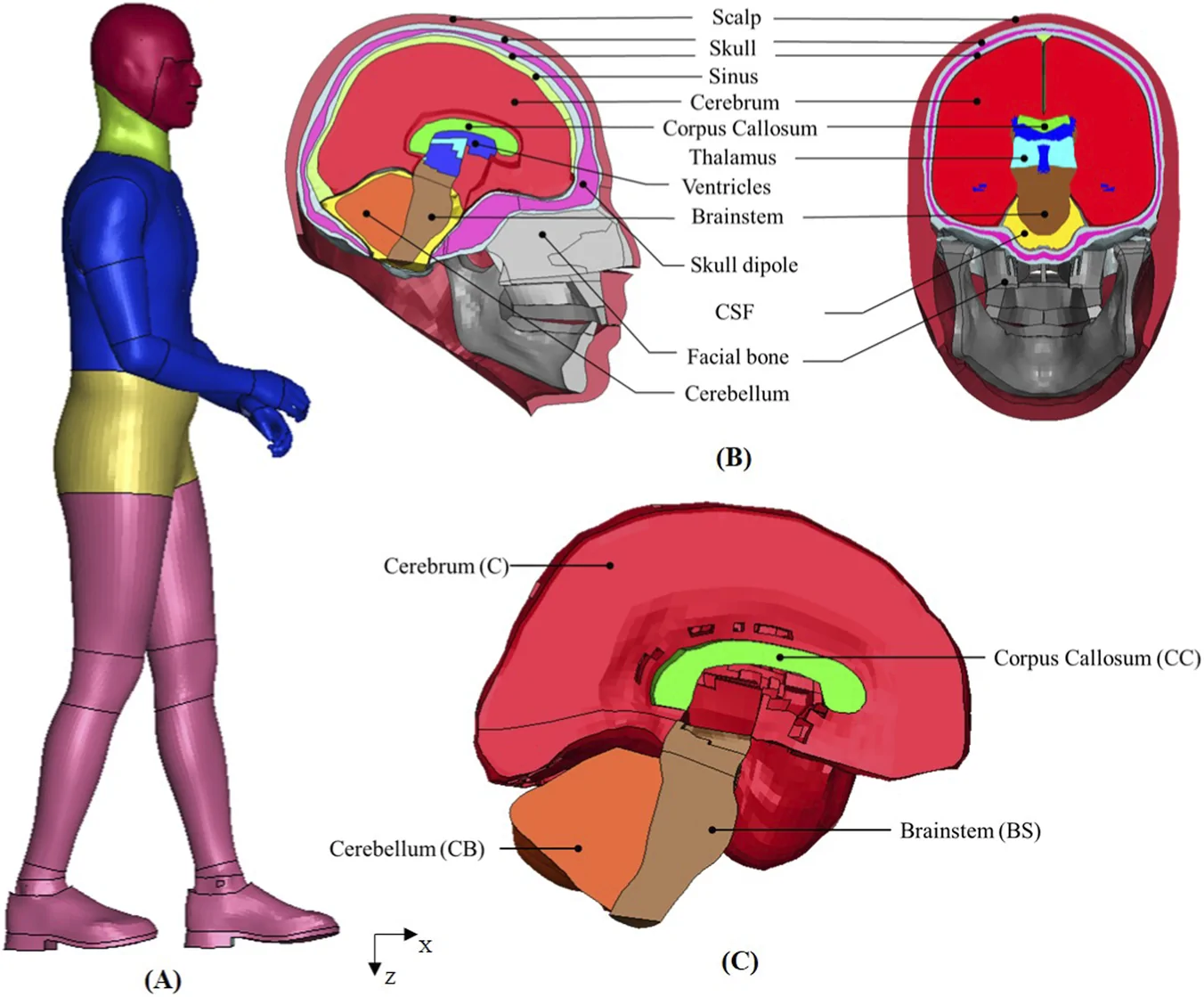
**(A)** Three-dimensional full body finite element human model. **(B)** Head model depicting various anatomical components (scalp, skull, sinus, cerebrum, corpus callosum, thalamus, ventricle, brainstem, skull diploë, CSF, facial bone, and cerebellum). **(C)** Brain region of interest (ROIs) considered for region specific analysis.

This study utilized a full-body human computational model based on the Global Human Body Models Consortium (GHBMC). We used a detailed pedestrian model, M50-P, version 1.5 of GHBMC (Figure 1A). The full-body model served two important purposes: it captured blast wave reflections from the full body, and it also provided a biofield neck, which was critical for capturing head kinematics in response to blast loading. The full-body human model (including head model) is extensively validated (Gayzik et al., 2018; Gayzik et al., 2011; Hostetler and Gayzik, 2024; Mao et al., 2013; Zaseck et al., 2016) under blunt impact loading through a multi-institutional effort. Sutar et al. (Sutar and Ganpule, 2024) validated a similar head model (Figure 1B) at IOPs of 70 kPa, 140 kPa, and 210 kPa, which fall within the lower intensities considered in this study. The head model was validated against skull surface pressures, brain pressures, and head kinematics. We describe the details of the head model in the following section, as the focus of this work is blast induced brain injury.

The anatomically accurate head model (Figure 1B) was based on the MRI and CT images of a 50th percentile healthy male volunteer (age = 26 years, weight = 78.6 kg, height = 174.9 cm) (Mao et al., 2013). It consisted of the facial bones, scalp, three layered skull, dura, pia, arachnoid, CSF, and the brain (Figure 1B). The brain included all major substructures such as the cerebrum, cerebellum, brainstem, thalamus, falx, tentorium, ventricles, corpus callosum, and the basal ganglia. The head model was meshed with 270,552 hexahedral elements, with the brain comprising 156,091 elements. The skull and facial bones were modeled as elastic plastic. The brain and scalp were modeled as linear viscoelastic; all other structures were modeled as linear elastic. The material properties are listed in Table 1. Note that the same material properties as that of the validated model (Sutar and Ganpule, 2024; Mao et al., 2013) were adopted. The choice of the constitutive model and model parameters was consistent with the literature (Mao et al., 2013).

**TABLE 1:** Material properties of various components of the head model (Mao et al., 2013).

Component	Density (kg/m^3^)	Elastic modulus (GPa)	Yield stress (MPa)	Tangential modulus (MPa)	Poisson’s ratio
Skull	2100	10	90	500	0.25
Skull diploë	1000	0.6	4	20	0.3
Component	Density	Bulk modulus (GPa)	Short-term shear modulus (kPa)	Long-term shear modulus (kPa)	Decay constant s^-1^
Cerebrum gray matter, cerebellum, brainstem, thalamus, basal ganglia	1060	2.19	6	1.2	80
CSF, 3rd and later ventricle	1040	2.19	0.5	0.1	80
Corpus callosum Cerebrum white matter	1060	2.19	7.5	1.5	80
Facial tissue	1100	0.005	340	140	33.33
Scalp	1100	0.02	1700	680	33.33
Component	Density (kg/m^3^)		Young’s modulus (GPa)		Poisson’s ratio
Skin	1100		0.01		0.45
Dura	1100		0.0315		0.35
Falx, Pia	1100		0.0125		0.35
Arachnoid	1100		0.012		0.35
Tentorium	1100		0.0315		0.3

In this work, we considered four brain regions as regions of interest (ROIs): the cerebrum (C), cerebellum (CB), corpus callosum (CC), and brainstem (BS) for biomechanical analysis (Figure 1C). The number of elements and volumes of the selected ROIs are 46,040 and 892.15 cm^3^ for the cerebrum, 14,280 and 115.67 cm^3^ for the cerebellum, 980 and 19.70 cm^3^ for the corpus callosum, and 2,280 and 20.88 cm^3^ for the brainstem, respectively. These regions were selected for their critical roles in the structural and functional aspects of the brain. Furthermore, these regions have been shown to be vulnerable to mild bTBI in clinical investigations (e.g., see (Hayes et al., 2015; Mac Donald et al., 2013; Mu et al., 2017)).

### 2.2 Computational modeling of blast wave head interaction

**FIGURE 2:**
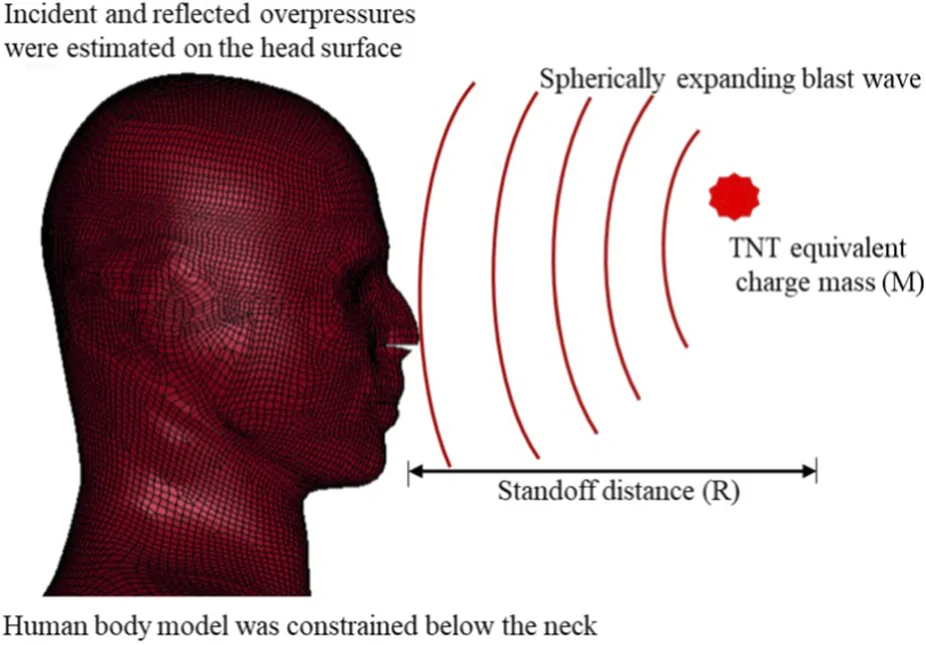
Schematic depicting the interaction of the blast wave with the human body using the Conventional Weapon (ConWep) technique. In the ConWep technique, explicit modeling of the air domain was not required. Instead, incident and reflected overpressures were determined on the head surface using Kingery-Bulmash equations (Kinney and Graham, 2013).

In the present study, blast wave head interactions were modeled using the ConWep technique (Figure 2), a computationally efficient approach for simulating primary blast effects (Sutar and Ganpule, 2024; Panowicz et al., 2017; Yu and Ghajari, 2019). ConWep represents blast wave propagation in an infinite domain and estimates the resulting overpressure-time histories on the target structure, in this case, the human head. The imposed overpressure-time histories were derived from empirical equations developed by Kingery and Bulmash. These equations were obtained by fitting extensive data from controlled free field air blast experiments (Swisdak, 1994; Jeon et al., 2017; Sutar and Ganpule, 2021). These empirical relations were implemented in the Conventional Weapons (ConWep) program (Ngo et al., 2004).

In the ConWep technique, the charge mass (M) and standoff distance (R) were specified (Figure 2). The resulting incident and reflected overpressure profiles on the target were estimated using empirical equations (Jeon et al., 2017; Sutar and Ganpule, 2021; Sutar and Ganpule, 2022; Kinney and Graham, 2013). This enabled the estimation of incident and reflected overpressure profiles without requiring the explicit modeling of the explosive charge and the surrounding air domain (so-called coupling approach), thereby drastically reducing the computational cost. We used ConWep because we simulated a large number (i.e., seventy-two) of blast overpressure and duration combinations as input. Further, each simulation was run for a longer duration of 80 ms. Explicitly modeling the air domain for such a duration typically requires ∼100 h of CPU time on 32 processors (Intel Xeon Gold, processor speed 2.3 GHz, 4 GB memory per processor). Sutar et al. (Sutar and Ganpule, 2024; Sutar and Ganpule, 2022) compared the ConWep approach with a fully coupled fluid-structure interaction technique (coupling approach) for predicting the biomechanical responses of the head/brain. They found that, when compared to the coupling method, the ConWep approach produced comparable predictions for the human head. This agreement supported the suitability of ConWep for the present study, where the primary objective was to evaluate brain biomechanical responses rather than simulate detailed blast flow physics.

### 2.3 Simulated blast conditions

**FIGURE 3:**
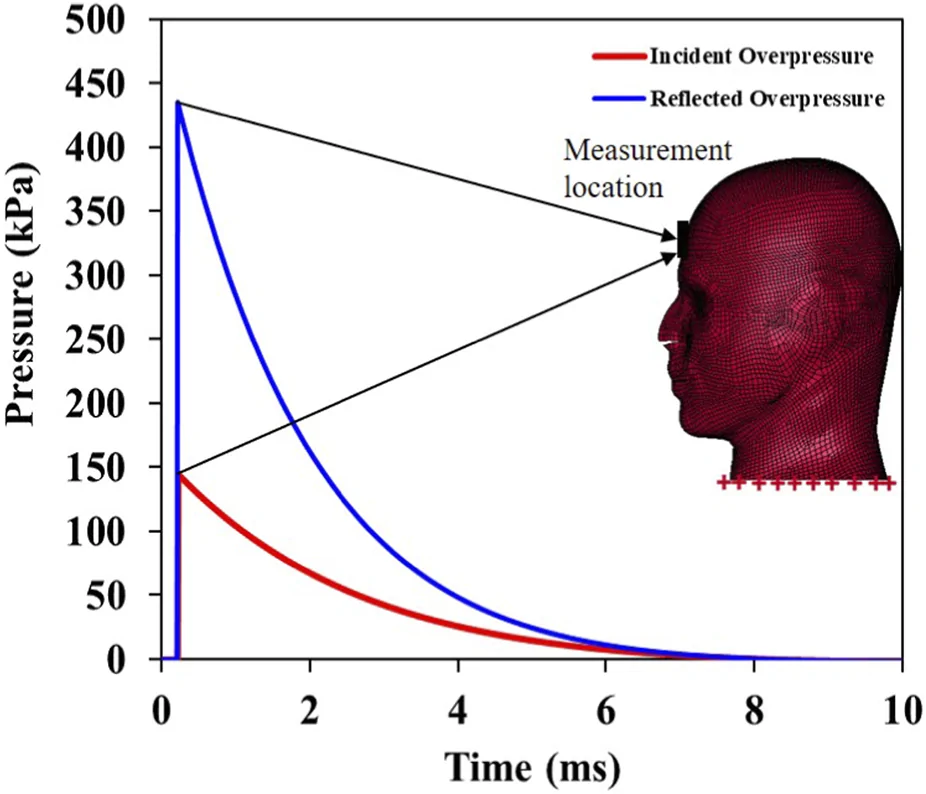
Representative incident and reflected overpressure time histories on the surface of the human head for the front orientation. As the incident wave impinged on the head, the pressure was amplified by 3.1 times due to the conversion of kinetic energy into potential energy. The insert shows the location for the plotted overpressures.

In this work, free-field blast conditions (Kinney and Graham, 2013) (Figure 3) were simulated. We considered peak incident overpressure (IOP) and positive phase duration (PPD) combinations corresponding to mild bTBI (Table 2). To represent the mild blast loading regime, we relied on animal studies, which remain the primary source for establishing injury thresholds. Mishra et al. (Mishra et al., 2016) conducted a series of experiments in rodents to identify bTBI thresholds. They suggested a peak incident overpressure range of 85–145 kPa for mild bTBI. Rafael et al. (Denny et al., 2021; Rafaels et al., 2012) conducted experiments in rodents to establish bTBI thresholds and scaled the results to humans using a mass scaling. They suggested a peak incident overpressure of 144 kPa corresponding to the mild bTBI threshold in humans. In another study on rats, they found no BBB leakage at IOPs below 70 kPa (Kuriakose et al., 2018). Therefore, 70 kPa was included as a conservative lower bound in this study. Panzer et al. (Panzer et al., 2014) studied brain responses in five brain models, ranging in size from mouse to human, across a wide range of blast loading. For incident overpressure, they obtained a scaling factor of 1.0 for scaling from rodent to human. Guided by these findings, we selected a peak incident overpressure (IOP) range of 70–145 kPa and the positive phase duration (PPD) range of 2–8 ms (Table 2). The range of PPD was based on PPD durations seen in realistic, far field IED blasts that are the primary source for mild bTBI in theatre (Ouellet and Petel, 2017; Denny et al., 2023; Chandra et al., 2017; Sundaramurthy and Chandra, 2014). Peak IOP and PPD were increased in steps of 10 kPa and 2 ms, respectively. As the ConWep formulation required charge mass (M) and stand-off distance (R) as inputs, we estimated these parameters corresponding to the desired peak IOP and PPD using the Kingery-Bulmash equations (Table 2) (Jeon et al., 2017), which were implemented in an in-house MATLAB code. The charger mass and standoff distance combinations were carefully selected to represent the relevant blast loading conditions of operational explosive threats (Ouellet and Petel, 2017; Sundaramurthy and Chandra, 2014; Panzer et al., 2012). For each loading case (Table 2), simulations were performed for both frontal and side orientations. The analysis was done based on a total of 72 cases (i.e., 36 per orientation).

**TABLE 2:** Simulated blast loading conditions corresponding to mild bTBI (Mishra et al., 2016; Rafaels et al., 2012). For the desired peak IOP and PPD, the corresponding values of charge mass (M) and standoff distance (R) were calculated using Kingery-Bulmash equations (Kinney and Graham, 2013).

Peak incident overpressure (IOP) (kPa)	Positive phase duration (PPD) (ms)	Standoff distance (R) (m)	TNT equivalent charge mass (M) (kg)
70	2	2.32	0.368
70	4	4.6	2.86
70	6	7	10.1
70	8	9.3	23.7
80	2	2.25	0.41
80	4	4.5	3.25
80	6	6.7	10.82
80	8	9	26.2
90	2	2.3	0.53
90	4	4.4	3.64
90	6	6.6	12.27
90	8	8.8	29.1
100	2	2.2	0.53
100	4	4.3	3.95
100	6	6.5	13.65
100	8	8.6	31.6
110	2	2.2	0.607
110	4	4.3	4.53
110	6	6.4	14.93
110	8	8.5	35
120	2	2.2	0.686
120	4	4.3	5.12
120	6	6.4	16.9
120	8	8.5	39.55
130	2	2.2	0.767
130	4	4.3	5.73
130	6	6.4	18.9
130	8	8.5	44.2
140	2	2.2	0.85
140	4	4.3	6.35
140	6	6.5	21.93
140	8	8.6	50.8
145	2	2.2	0.893
145	4	4.3	6.67
145	6	6.5	23
145	8	8.6	53.31

### 2.4 Boundary conditions

In the current study, the full-body model was made completely constrained below the neck (see Figure 2). This condition depicted a few real life combat scenarios in which the personnel’s portion below the neck was not exposed to the blast (e.g., standing behind a wall or in a vehicle, in the breaching operations). The rationale for adopting this boundary condition was further supported by our previous study (Sutar and Ganpule, 2024). We compared brain biomechanical responses under constrained and unconstrained conditions in detail. The finding revealed that under constrained and unconstrained conditions, brain pressure remained unaffected (∼200 kPa), whereas the equivalent stress response deviated after ∼20 ms, with a higher magnitude in the constrained condition (Supplementary Figure S1). Since the magnitude of the equivalent stress was higher (by ∼250%) in the constrained case, we considered the constrained case scenario as the worst-case scenario and used this condition for the rest of the simulations. The comparative pressure-time and equivalent stress-time histories for constrained vs. unconstrained boundary conditions are in the Supplementary Material (Supplementary Figure S1).

### 2.5 Solution scheme

Nonlinear, transient, dynamic analysis was performed using an explicit scheme and a massively parallel processing solver in LS-Dyna. The total simulation time of 80 ms was considered, as both volumetric and deviatoric responses were played out and fully established by this time. A typical simulation required ∼12 h of CPU time on 32 Intel Xeon Gold processors (processor speed: 2.3 GHz, 4 GB of memory per processor).

### 2.6 Analysis of brain biomechanical responses and data reduction

To evaluate brain tissue responses, we extracted element-wise time histories of pressure, equivalent stress (von Mises), maximum principal strain (MPS), and maximum principal strain rate (MPSR). These biomechanical parameters were chosen because they have been associated with neurological impairments, such as concussion, axonal damage, white matter injury, and neuronal injury (Meaney et al., 2014; Bar-Kochba et al., 2016; McAllister et al., 2012; Giordano et al., 2014; Ho and Kleiven, 2007; Zhang et al., 2004). The peak values of each of the aforementioned biomechanical parameters at each element were obtained. Note that the peak values were independent of the time at which the peak occurred. The 95th percentile values of the aforementioned biomechanical parameters were calculated using the element-wise peak values. The 95th percentile analysis was performed at the global (whole brain) level and substructure wise for each ROI (for details, refer to Section 2.1) (Figure 1C) across all simulations.

The estimated 95th percentile values form the basis for data analysis. To further characterize the brain biomechanical parameters associated with mild bTBI, we implemented descriptive statistical analysis, which included the minimum, maximum, mean, and 25th −75th percentile interquartile range. Normality was tested for each brain biomechanical parameter using the Lilliefors test. Because the responses were normally distributed, an ANOVA was performed to assess concentration within a given ROI. Additionally, when significant differences were observed, pairwise comparisons were conducted using a two-sample t-test. To account for multiple comparisons, p-values were adjusted using the Bonferroni method. The purpose of the statistical analysis was only to provide a quantitative comparison of relative differences in biomechanical responses across ROIs, rather than to make any biological inference.

Regression analysis was used to assess the relationship between brain biomechanical parameters (i.e., pressure, equivalent stress, MPS, and MPSR) and blast parameters (i.e., peak IOP, PPD, and impulse). The coefficient of determination (*r* ^2^) was determined for each regression as a goodness of fit.

Data analyses were performed using custom MATLAB scripts in conjunction with the built-in statistical toolbox features of MATLAB (MathWorks, 2019a). A significance level ( α ) of 0.05 was set *a priori* for each statistical test.

## 3 Results

The trends/findings of the results presented in this section are valid for all simulated cases, unless stated otherwise.

### 3.1 Interaction of the blast wave with the human head

**FIGURE 4:**
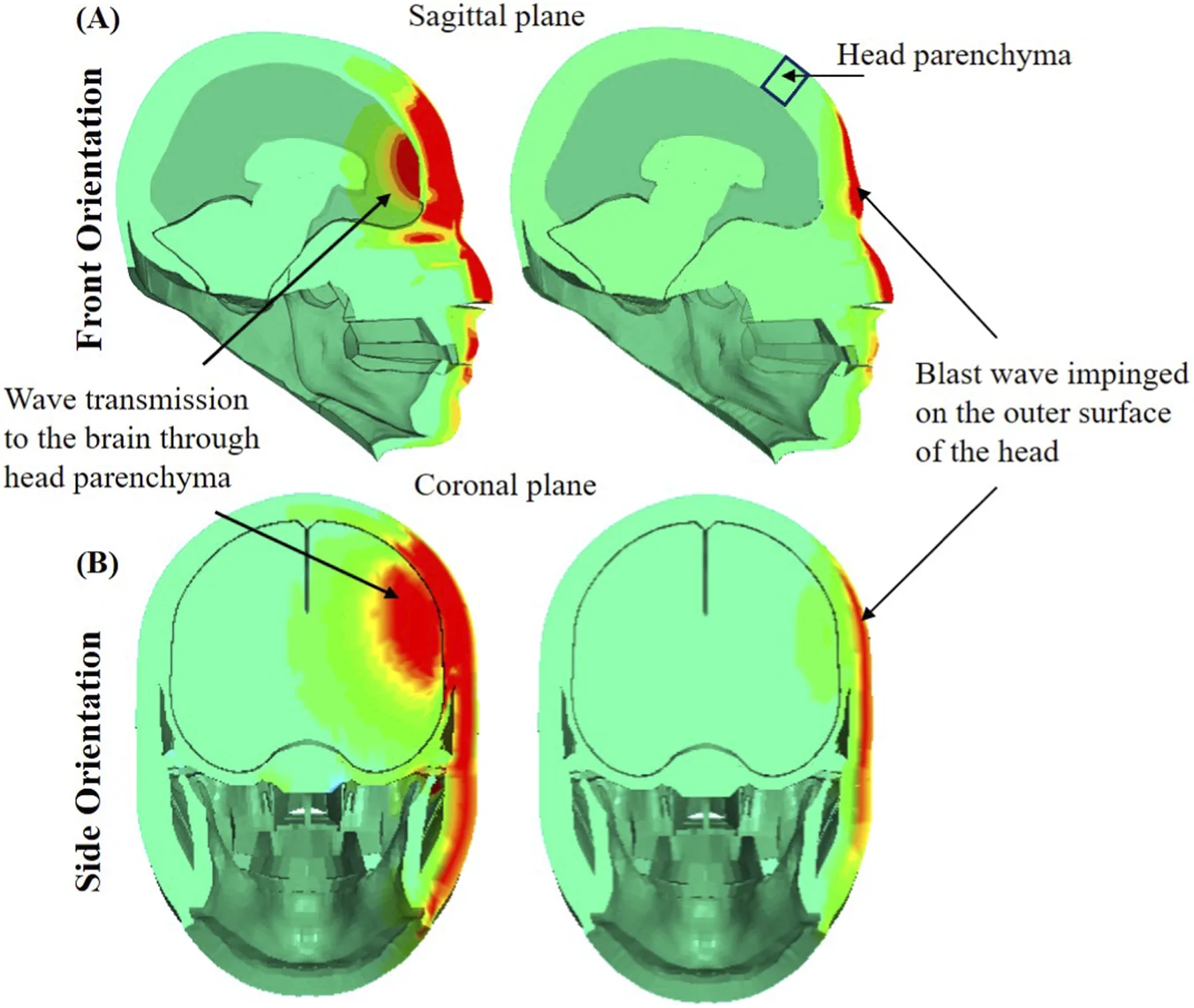
Picture depicting a wave propagation through the head parenchyma for **(A)** front orientation (represented in sagittal plane), **(B)** side orientation (represented in coronal plane). The blast wave is transmitted to the brain as a result of wave propagation through the head parenchyma.

**FIGURE 5:**
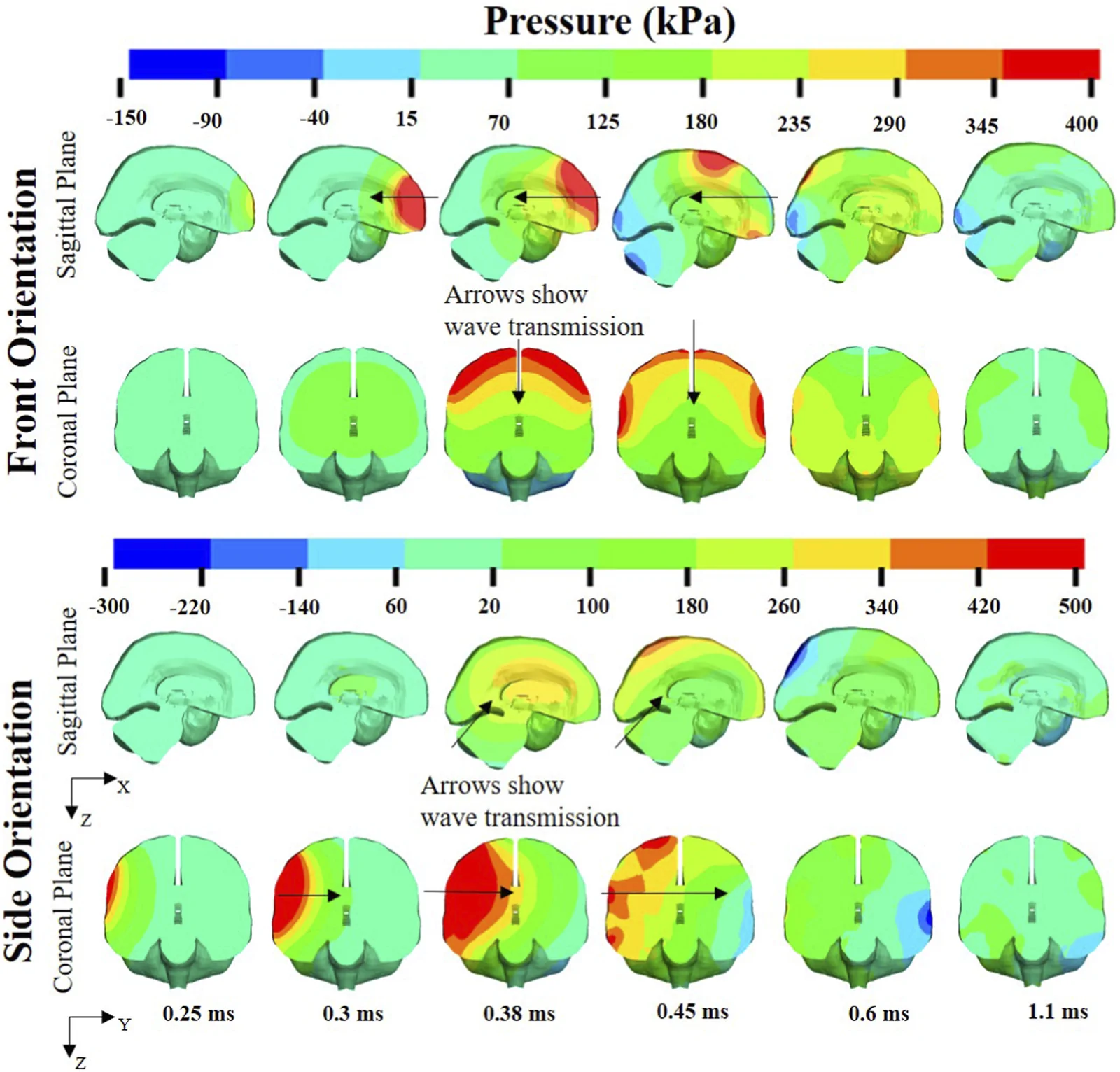
Spatiotemporal evolution of brain pressure. Wave propagation is played out in ∼1 ms. Arrows depict the wave transmission in the brain.

**FIGURE 6:**
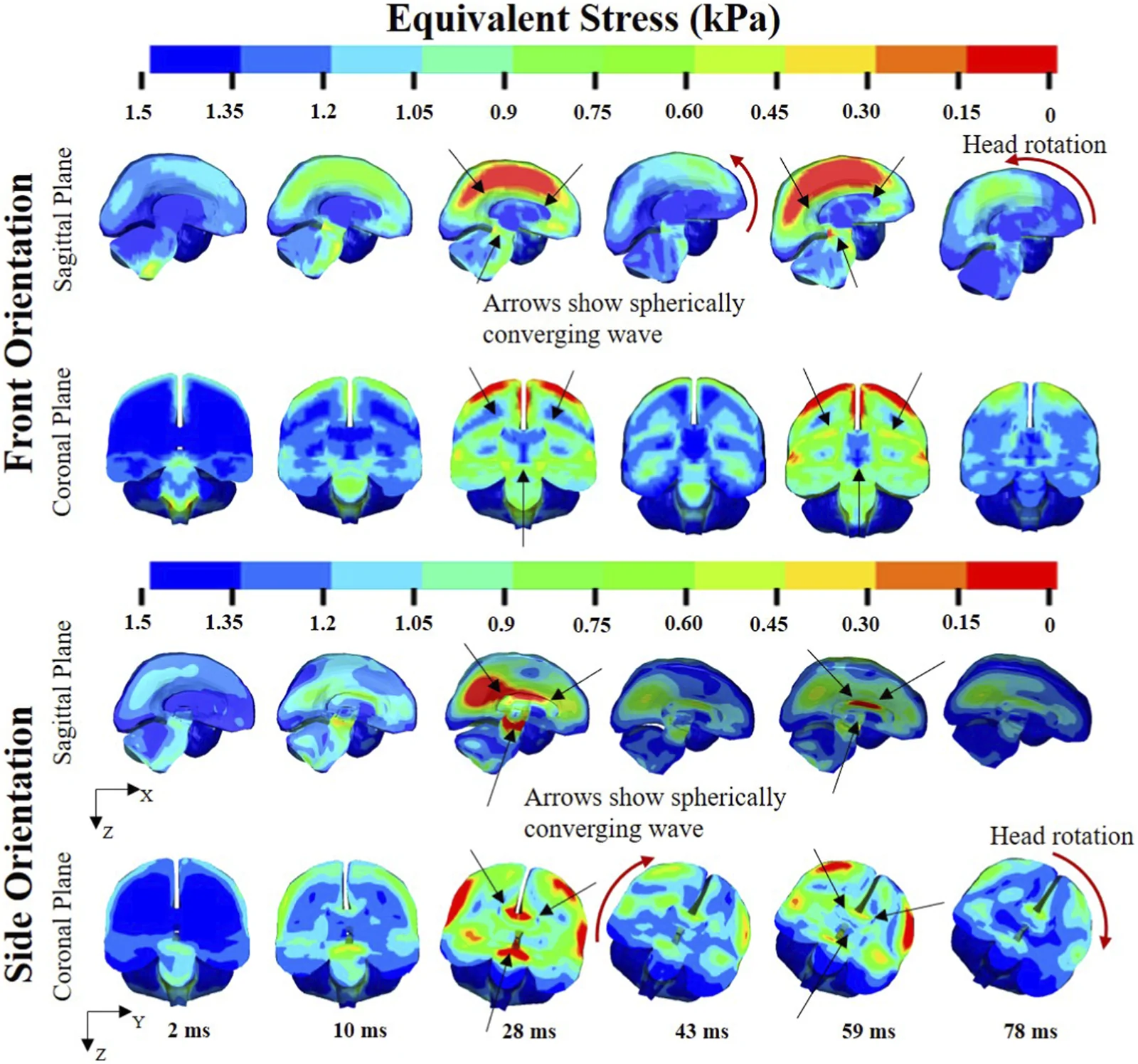
Spatiotemporal evolution of equivalent stress in the brain. Wave propagation is played out in ∼80 ms. The temporal distribution of the equivalent stress wave exhibits a spherically converging pattern, as depicted by the black arrows. The equivalent stress evolution is governed by the head rotation, as depicted using the red arrows.

Figure 3 shows a representative incident and reflected overpressures at the head surface for the front orientation. As the blast wave impinged on the head, the pressure on the surface of the head was amplified due to the conversion of kinetic energy contained in a blast into potential energy. This amplified pressure is referred to as the reflected overpressure (ROP). The loading on the surface of the head initiated a wave propagation in the head parenchyma, leading to a transmitted wave in the brain tissue (Figure 4). The transmission produced volumetric (bulk, Figure 5) and shear (deviatoric, Figure 6) waves in the brain tissue. Shear waves were generated due to the reflection of the wave from the skull-brain boundaries. The volumetric response in the brain had a timescale of a few milliseconds (∼1 ms) and controlled the evolution of pressure (Figure 5). The deviatoric response had a timescale of ∼80 ms and exhibited a spherically converging pattern (Figure 6). The deviatoric response governed the evolution of equivalent stress. Note that there was a timescale separation between volumetric and deviatoric responses, which were governed by two distinct mechanisms wave propagation and inertial, respectively.

### 3.2 Correlation and sensitivity of brain biomechanical parameters to the blast parameters

The effects of the peak incident overpressure (IOP), positive phase duration (PPD), and blast impulse on brain biomechanical parameters were investigated.

Tables 3, 4, respectively, tabulate the representative results of brain biomechanical responses as a function of peak IOP (PPD constant) and PPD (peak IOP constant). Biomechanical responses for all simulated cases are presented in the Supplementary Table S1 and avoided in the main manuscript for brevity. Pressure, equivalent stress, MPS, and MPSR increased by ∼13% as the peak IOP was increased in steps of 10 kPa (Table 3). When the PPD was increased (Table 4) from 2 to 8 ms (in 2 ms increments), pressure and MPSR increased by 1%–5% and 6%–19%, respectively. On the contrary, equivalent stress and MPS were increased, respectively, by 46%–112% and 42%–108%.

**TABLE 3:** 95th percentile values of the brain biomechanical parameters for the whole brain as a function of peak IOP (70–145 kPa) for a constant PPD of 2 ms.

Peak IOP (kPa)	Front orientation				Side orientation			
	Pressure (kPa)	Equivalent stress (kPa)	MPS (%)	MPSR (s^-1^)	Pressure (kPa)	Equivalent stress (kPa)	MPS (%)	MPSR (s^-1^)
70	138.1	0.089	2.11	25.1	184.6	0.112	2.68	20.2
80	159.72	0.0982	2.34	28.41	217	0.126	3	23.2
90	186.26	0.115	2.71	32.5	255.2	0.146	3.5	26.8
100	207	0.12	2.88	35.2	285.2	0.155	3.7	29.4
110	232.26	0.135	3.2	38.7	321.4	0.17	4.1	32.7
120	258.17	0.149	3.5	42.33	358.6	0.186	4.4	36.1
130	285.86	0.164	3.86	46	395.1	0.194	4.58	39.2
140	312.3	0.18	4.2	49.67	436.1	0.222	5.3	43.1
145	327	0.19	4.45	51.5	455.8	0.225	5.3	44.9

**TABLE 4:** 95th percentile values of the brain biomechanical parameters for the whole brain as a function of PPD (2–8 ms) for a constant peak IOP of 145 kPa.

PPD (ms)	Front orientation				Side orientation			
	Pressure (kPa)	Equivalent stress (kPa)	MPS (%)	MPSR (s^-1^)	Pressure (kPa)	Equivalent stress (kPa)	MPS (%)	MPSR (s^-1^)
2	327	0.19	4.5	51.5	455.8	0.225	5.3	44.9
4	344.74	0.402	9.2	61.2	479.3	0.425	10	51.4
6	354.5	0.662	15.2	67.1	487.6	0.645	14.5	55
8	359.2	0.964	21.6	71	495.7	0.88	18.9	57.5

**FIGURE 7:**
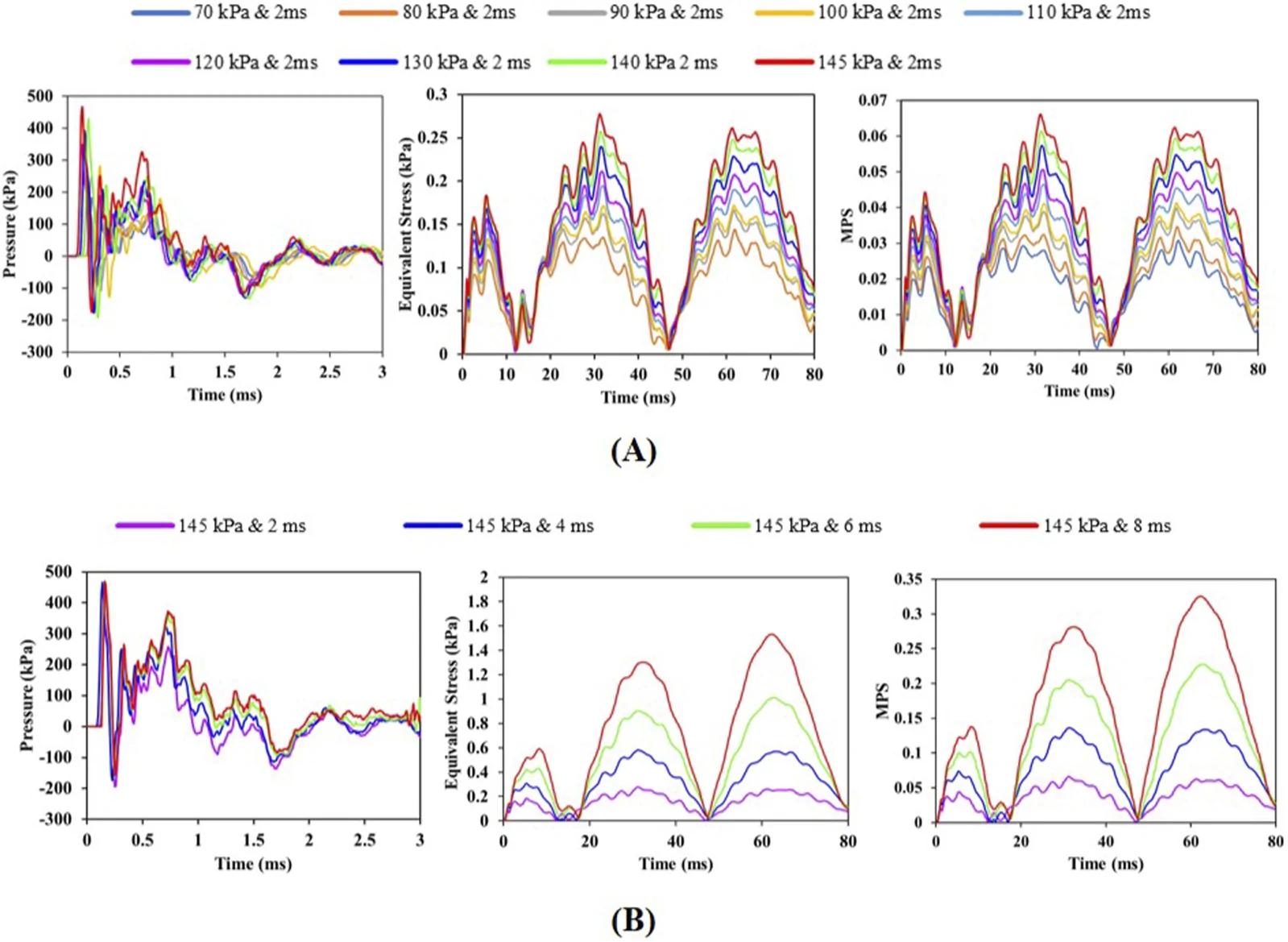
Temporal evolution of brain biomechanical parameters (pressure, equivalent stress, and MPS) as a function of peak IOP and PPD. Representative results for **(A)** change in peak IOP (70–145 kPa) for a constant PPD of 2 ms, **(B)** change in PPD (2–8 ms) for a constant peak IOP of 145 kPa. Figures show that the shape of biomechanical responses remains unchanged with the change in IOP or PPD.

When brain biomechanical parameters were compared with the impulse, the subtleties of their dependencies on blast parameters were more evident. Linear regression analysis between impulse and blast parameters yielded *r* ^2^ values of 0.25 for brain pressure, 0.57 for MPSR, and 0.98 for both equivalent stress and MPS. For peak IOP, the *r* ^2^ values were 0.98 for brain pressure, 0.78 for MPSR, and 0.2 for equivalent stress and MPS. Thus, pressure and MPSR were better correlated with the peak IOP, whereas equivalent stress and MPS were better correlated with impulse. Note that the shape (i.e., temporal evolution) of the brain biomechanical parameters remained unchanged with the change in peak IOP and PPD (Figure 7). A similar trend, as stated above, was observed across all ROIs. Among the ROIs, C and CC exhibited a greater sensitivity to PPD.

### 3.3 Concentration of brain biomechanical parameters among ROIs

**FIGURE 8:**
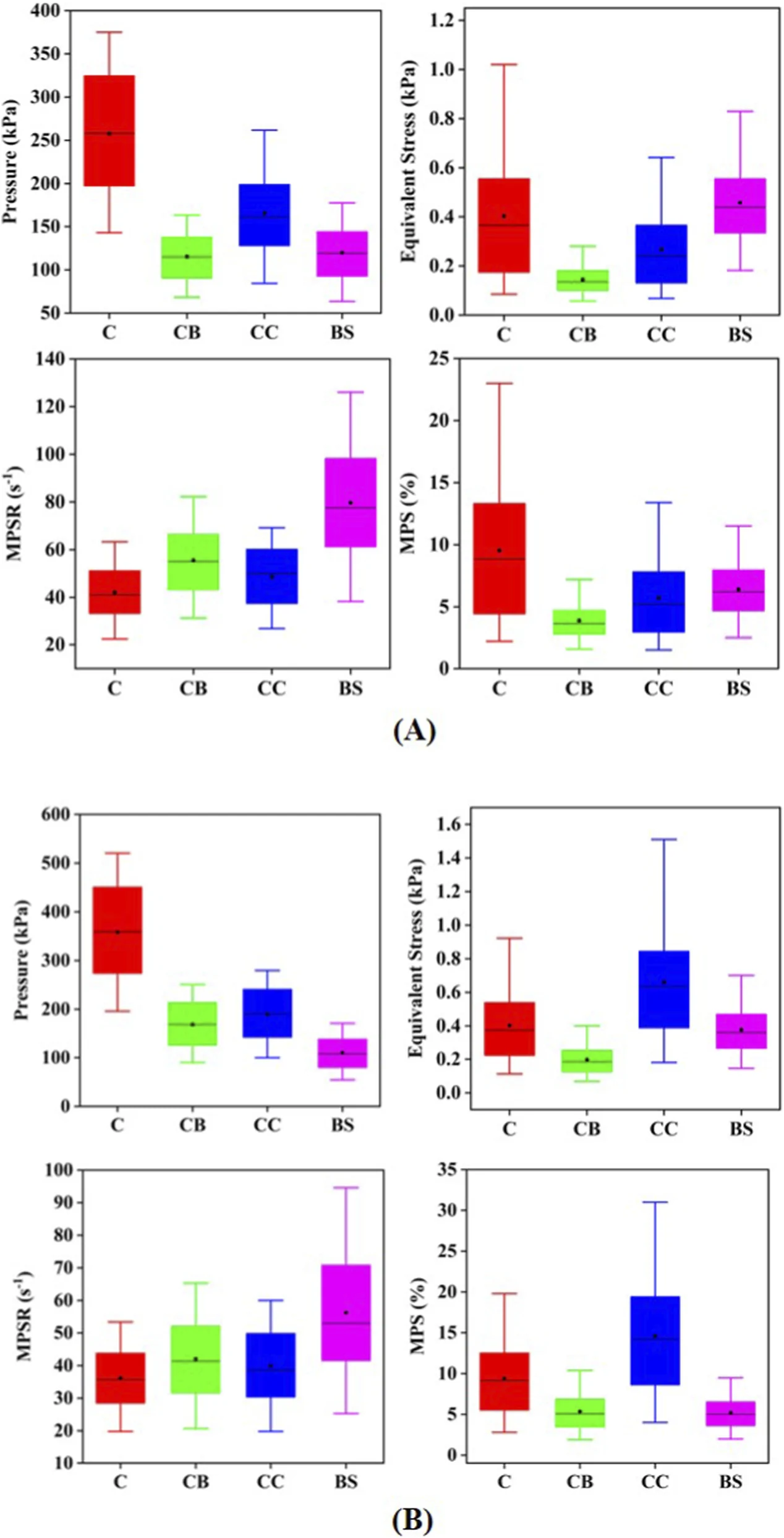
Concentration of brain biomechanical parameters among brain ROIs. **(A)** front orientation **(B)** side orientation. In the box plots, the black dot represents the mean, the central black line indicates the median, and the box bounds correspond to the 25th and 75th percentiles.

Figure 8 shows the distribution of brain biomechanical parameters within each ROI (as stated in the method section). To identify which part of the brain was most affected by each parameter, a *post hoc* two-sample t-test was employed. For the frontal orientation (Figure 8A), the pressure was most concentrated in the C, whereas CB and BS were least affected. Equivalent stress was concentrated in C and BS, while CB showed the least sensitivity. The peak concentration of MPS was found in the C, followed by CC and BS, whereas CB was the least affected. MPSR was concentrated in BS; CC, CB, and C showed statistically similar responses.

For the side orientation (Figure 8B), a similar pattern in ROI concentration was observed for pressure and MPSR. Equivalent stress was found to be most concentrated in CC, followed by C and BS, while CB showed the least sensitivity. MPS was most concentrated in CC, followed by C, and the least in CB and BS.

### 3.4 Mean and interquartile values of brain biomechanical parameters

Tables 5, 6 tabulate the values of brain biomechanical parameters corresponding to simulated blast conditions representing mild bTBI. The tabulated values were represented as the mean and 25th-75th percentile interquartile range for the whole brain and for each ROI (Figure 1C). Note that the values were based on 36 simulations for each orientation (for details, see Supplementary Table S1). In general, based on the obtained values, side orientation (Table 6) yielded a more deleterious response compared to front orientation (Table 5), except for MPSR. The values of brain biomechanical parameters between the whole brain and C were similar, as C accounts for 41% of the whole brain by volume. Analysis among ROIs revealed that the highest values of pressure (257.9 kPa for the front orientation and 358 kPa for the side orientation) were observed in the C. The maximum values of equivalent stress and MPS were found in C (0.4 kPa and 9.5%) and CC (0.67 kPa and 14.6%) for front and side orientations, respectively. The highest MPSR values, 79.7 s^-1^ for front orientation and 56.2 s^-1^ for side orientation, were found in BS.

**TABLE 5:** Mean and 25th – 75th interquartile values of brain biomechanical parameters for the whole brain and various ROI for simulated blast loading conditions for the front orientation.

Biomechanical parameters Mean and (25^th^-75^th^)	Region of interest (ROI)				
	Whole brain	Cerebrum (C)	Cerebellum (CB)	Corpus callosum (CC)	Brainstem (BS)
Pressure (kPa)	247.4 (191.3–310.3)	257.9 (199.3–323.7)	115.2 (92.1–137.1)	165.5 (129.2–196.1)	119.8 (92.9–143.9)
Equivalent stress (kPa)	0.4 (0.19–0.52)	0.40 (0.18–0.54)	0.14 (0.1–0.18)	0.27 (0.13–0.36)	0.46 (0.34–0.55)
Maximum Principal strain (MPS)	9% (4%–12%)	9.5% (5%–13%)	3.9% (3%–5%)	5.7% (3%–8%)	6.4% (5%–8%)
Maximum Principal strain rate (MPSR) (s^-1^)	47.2 (38.5–56.2)	42 (33.5–50.5)	55.5 (43.3 66.1)	48.6 (38–60.1)	79.7 (61.8–96.9)

**TABLE 6:** Mean and 25th – 75th interquartile values of brain biomechanical parameters for the whole brain and various ROI for simulated blast loading conditions for the side orientation.

Biomechanical parameters Mean and (25^th^-75^th^)	Region of interest (ROI)				
	Whole brain	Cerebrum (C)	Cerebellum (CB)	Corpus callosum (CC)	Brainstem (BS)
Pressure (kPa)	338.5 (260.3–424.6)	358 (275.8–450)	168.5 (128–212.3)	190 (144.1–240)	110 (81.4–137.2)
Equivalent stress (kPa)	0.4 (0.22–0.51)	0.40 (0.22–0.53)	0.2 (0.13–0.25)	0.67 (0.4–0.87)	0.38 (0.27–0.47)
Maximum Principal strain (MPS)	9% (5.3%–11.7%)	9.4% (5.6%–12.4%)	5.3% (3.5%–6.8%)	14.6% (8.7%–19.2%)	5.2% (3.7%–6.5%)
Maximum Principal strain rate (MPSR) (s^-1^)	38.3 (30–46)	36.2 (28.7–43.1)	42 (32–51.3)	40 (30–49.5)	56.2 (41.8–70)

## 4 Discussion

Blast induced traumatic brain injury (bTBI) has been a critical concern in modern warfare (Tanielian et al., 2008; Warden, 2006). Over the years, considerable progress has been made in understanding the biomechanics of bTBI. However, the relationship between the external blast parameters and the internal brain response, especially in humans, is not fully known. Specifically, the sensitivity of the brain regional responses and associated human brain biomechanical responses with respect to the external blast parameters implied in mild bTBI is unavailable. In this work, we investigated the aforementioned aspects using the 3D computational model of the human head.

The reflected pressure (Figure 3) on the surface of the head due to the blast initiated a loading of the head parenchyma (Figure 4). The resulting biomechanical cascade (Figures 5, 6) within the human brain was broadly categorized into wave propagation (Figure 5) and the inertial (i.e., head acceleration) effect (Figure 6). Each of these effects had a distinct timescale (∼1 ms and ∼80 ms, respectively) and induced a distinct biomechanical signature in the brain. The temporal evolution of pressure within the brain was governed by wave propagation. The temporal evolution of equivalent stress and MPS was governed by the inertial effect. Since the inertial effect was similar to the blunt impact, the equivalent stress and MPS spatiotemporal patterns were similar to those observed in blunt impact (e.g. (Chen and Ostoja-Starzewski, 2010; Hardy, 2007; Ji et al., 2022),). Spatial evolution of equivalent stress and MPS showed a spherically converging (Chen and Ostoja-Starzewski, 2010; Zhao and Ji, 2020) pattern (Figure 6), whereas temporal evolution (Figure 7B) was sinusoidal (Hardy, 2007; Zhao and Ji, 2020). The pressure pattern within the brain, however, was different from the blunt impact. Specifically, the pressure time histories were played out within a very short time and followed a Friedlander waveform (Figure 7A), as opposed to a sinusoidal waveform in blunt impact (Hardy, 2007; Nahum et al., 1977). These findings regarding blast wave head interactions are consistent with a previous work (Ganpule et al., 2013; Sutar and Ganpule, 2024) wherein they simulated the interaction of the blast wave with the human head. Our results underscore that the blast simulations should be performed for a longer duration to observe the full spectrum biomechanical cascade (Figure 4). Most of the investigations (e.g. (Panzer et al., 2012; Chafi et al., 2010; Chafi et al., 2011; Zhang et al., 2013; Tan et al., 2014; Moore et al., 2009)) simulating blast wave interactions simulate blast response until 5–10 ms and typically fail to fully capture the temporal evolution of equivalent stress and MPS.

The correlation between brain biomechanical parameters and blast parameters revealed an interesting paradigm that can be attributed to wave propagation and inertial effects described above. Pressure and MPSR in the brain were strongly correlated with peak IOP (*r* ^2^ = 0.98 and 0.78, respectively), which may be attributed to their sensitivity to transient wave transmission and high loading rate during the initial blast interaction. In contrast, equivalent stress and MPS in the brain were strongly correlated with blast impulse (*r* ^2^ = 0.98), suggesting that cumulative loading effects and momentum transfer over the positive phase duration play a larger role in governing tissue deformation responses. The regression results were based on 36 cases for each orientation (Section 3.2; Supplementary Table S1). Further, these findings can also be supported by the fact that brain pressure was played out within ∼1 ms (Figure 5), whereas equivalent stress and MPS were evolved over ∼80 ms (Figure 6).

We also observed that an increase in PPD had a more deleterious effect as compared to an increase in peak IOP (Figure 7). Pressure, equivalent stress, MPS, and MPSR increased by ∼13% as the peak IOP was increased in steps of 10 kPa (Figure 7A; Table 3) for the same PPD. On the contrary, equivalent stress and MPS were increased in the range of 42%–112% as PPD was increased in steps of 2 ms (Figure 7B; Table 4) for the same peak IOP. This clearly shows that for a given peak IOP, PPD governs equivalent stress and MPS (*r* ^2^ > 0.95 for each simulated peak IOP). This has significant implications for injury. Blast parameters (peak IOP and impulse) and corresponding brain biomechanical parameters (pressure, equivalent stress, MPS, MPSR) can induce distinct types of injury (i.e., phenotype) within the brain. For example, increased pressure within the brain may cause swelling and hematomas. Equivalent stress is postulated to be responsible for DAI, whereas MPS and MPSR are associated with axonal stretching.

The aforementioned findings regarding the correlation between blast parameters and brain biomechanical parameters are consistent with the literature. Panzer et al. (Panzer et al., 2012) conducted a numerical simulation using a 2D head model across a range of peak IOP and PPD combinations. They found that peak IOP was correlated with brain pressure, whereas impulse was correlated with equivalent stress and strain in the brain. Hue et al. (Hue et al., 2016) performed bTBI investigation using cultured mouse brain blood brain barrier (BBB) cells and observed that the BBB breakage was strongly correlated with blast impulse rather than peak IOP. Reneer et al. (Reneer et al., 2014) observed greater BBB disruption and surface hemorrhage in rats as PPD was increased, while keeping the peak IOP constant.

Compared with the front orientation, the side orientation produced higher mean and interquartile values for brain biomechanical parameters (Tables 5 and 6), except for MPSR. Pressure was higher in C, CC, and CB; equivalent stress and MPS were higher in CC and CB (Figure 8). Increased pressure in the side orientation could be attributed to the larger cranial lateral surface area (590.5 cm^2^ for the side and 414.9 cm^2^ for the front) (Figure 4) and relatively lower skull thickness in the temporal region (∼4.1 mm in the side and ∼10.2 mm in the front). Other blast investigations using a computational human head model also found that the side orientation was more severe than the front orientation (Zhang et al., 2013; Duan et al., 2024; Hua et al., 2017; Salimi Jazi et al., 2016). Among all brain biomechanical parameters and ROIs, equivalent stress and MPS showed the largest increase (∼150% from front-to-side orientation) in the CC (Table 5 and 6). This could be attributed to the considerable shearing observed in central regions for side orientation (Figure 6). Clinical investigations (e.g. (Hayes et al., 2015; Mu et al., 2017; Venkatasubramanian et al., 2020; Ganpule et al., 2017)) suggest that central white matter structures (e.g., corpus callosum, corona radiata, corticospinal tract, and internal capsule) are the most vulnerable regions in bTBI.

Investigation of the concentration of biomechanical parameters across the brain ROIs revealed that the pressure was concentrated in the C, whereas the equivalent stress and MPS were concentrated in the CC (Figure 8). This spatial dependence of various biomechanical parameters (and associated injury phenotypes) can be attributed to the distinct mechanisms (i.e., wave transmission and inertial Figures 5, 6) governing the response of the brain to the blast. Susceptibility of C and CC to blast loading was also observed in other investigations, as explained below. C governs cognition, sensation, and voluntary motor function. Elevated pressure in this region may contribute to transient neural dysfunction, vascular disturbance, and blood-brain barrier (BBB) disruption. Such impairments had been observed in rodents and humans exposed to a blast (Kabu et al., 2015; Shively et al., 2016; Yeoh et al., 2013; Kawoos et al., 2016; Antunes and Biala, 2012; Aravind et al., 2020b).

CC serves as a conduit for information transfer between the left and right hemispheres of the brain; any damage to CC can impair information processing speed, attention, memory, and executive function. Clinical studies regularly report the aforementioned symptoms in military personnel suffering from mild bTBI (Sachdeva and Ganpule, 2024; Goldstein et al., 2012; Borinuoluwa and Ahmed, 2023; McGlinchey et al., 2017; Siedhoff et al., 2022; Giordano et al., 2014; Phipps et al., 2020). For example, Goldstein et al. (Giordano et al., 2014) observed reduced memory and attention, executive dysfunction, and motor deficits in returning soldiers. Various neuroimaging studies in veterans exposed to blast identified CC as the most affected brain region, based on the presence of diffusion (Mac Donald et al., 2011; Mu et al., 2017; Levin et al., 2010; Poliva et al., 2024). Shively et al. (Shively et al., 2016) investigated post-mortem brains from military personnel exposed to blast and demonstrated axonal damage in the cortical white matter and the CC. A vulnerability of CC was also observed in rodents in the form of axonal damage and microstructural abnormalities (Xu et al., 2016; Venkatasubramanian et al., 2020; Hernandez et al., 2018; Shetty et al., 2014). In this work, we have demonstrated ROI specific vulnerabilities (Figure 8) and provided associated biomechanical parameter values (Table 5 and 6) that will be useful in designing futuristic helmets and guiding FE simulation assisted clinical diagnostics.

In order to understand commonalities and differences between blast and blunt impact loading, we compared mean and interquartile values of brain biomechanical parameters corresponding to the simulated mild bTBI loading of this work (Table 5, 6) against Beckwith’s (Beckwith et al., 2018) mild blunt impact TBI data (Supplementary Table S2). This comparison is important from both a fundamental scientific standpoint and for devising effective mitigation strategies. Beckwith (Beckwith et al., 2018) conducted computational simulations to evaluate brain tissue response under blunt impact. They utilized measured head kinematics data from collegiate and high school American football players as input to the 3D finite element head model. From FE simulations of concussed and non-concussed cases, they derived median and 25th–75th percentile values (Supplementary Table S2) corresponding to mild blunt TBI. We specifically chose Beckwith’s data (Beckwith et al., 2018) because the authors have reported brain responses to mild blunt TBI, thus facilitating the desired comparison.

The mean and 25th–75th percentile values of pressure (247.4 kPa, 191.3–310.3 kPa) obtained in this work were high compared to those of Beckwith (67.12 kPa, 52.41–79.3 kPa). This is due to direct transmission (Figure 4) of the blast wave to the brain through the head parenchyma during the blast loading. Wave transmission is typically absent in the case of blunt impact (Hardy, 2007; Nahum et al., 1977). The mean and 25th–75th percentile values of rotational accelerations (5.78 krad/s^2^, 4.73–6.65 krad/s^2^) of this work were comparable to the values reported by Beckwith (4.364 krad/s^2^, 3.21–6.022 krad/s^2^). This was due to the fact that the rotational accelerations were governed by the inertial effects. MPSR values were also comparable and were of the order of 10^1^ s^-1^. This data suggests that blast is similar to the blunt impact, barring pressure transmission.

## 5 Limitation

This study has several limitations. We used the M50-P (version 1.5) male model of GHBMC, which does not account for inter-subject variability arising from differences in age, sex, material properties, and anatomy. The data presented in this work is based on the 50th percentile male. We considered only frontal and side orientations, whereas field blast exposures may involve more complex loading scenarios. Hence, angled orientations should be explored in the future. To simulate blast wave human body interactions, a computationally efficient ConWep approach was used in this work. Future studies, especially involving helmets and googles, should explore explicitly modeling the surrounding air domain and associated interaction effects with the head (e.g., underwash effect, flow separation, flow reunion (Ganpule et al., 2013; Sutar and Ganpule, 2022; Ganpule et al., 2012).

Note that in our previous study, we compared the ConWep and fluid-structure interaction (FSI) approaches and found that biomechanical responses were reasonably comparable between the two, especially in non-helmeted simulations (Sutar and Ganpule, 2024). In this work, we considered the human body fully constrained below the neck; other boundary conditions should be explored in the future.

Direct validation of the results presented in this work against human bTBI outcomes remains challenging due to the limited availability of such data in humans and in post-mortem human subjects (PMHS). Hence, overinterpretation of the data presented in this work, especially regarding the injury risk and clinical implications, should be avoided.

Overall, future studies should investigate anthropometric and sex variability, additional blast orientations, possible cases of blast plus blunt impact, and experimental investigations in humans and PMHS to facilitate qualitative and quantitative comparisons of simulation results.

## 6 Conclusion

This study investigated the relationship between external blast parameters and brain biomechanical parameters associated with mild bTBI using a 3D, computational human model. The key findings of this work are:

1. Blast wave induced a biomechanical cascade within the brain, which can be categorized into wave propagation and inertial effects (rotational head acceleration). Each of these effects had a peculiar timescale and induced distinct biomechanical signatures. Wave propagation within the brain governed the pressure, and inertial effects governed the equivalent stress and MPS.
2. The correlation between the blast parameters and brain biomechanical parameters revealed that brain pressure and MPSR were correlated with peak IOP, whereas equivalent stress and MPS were correlated with blast impulse. An increase in PPD had a more deleterious effect than an increase in peak IOP. These correlations were directly attributed to the biomechanical cascades of wave propagation and inertial effects.
3. Side orientation was found to be more severe than the front orientation in blast exposure, as the brain biomechanical responses measured in side orientation resulted in higher values of brain biomechanical parameters. Among the ROIs, the most affected region of the brain was the CC, as indicated by the highest equivalent stress and MPS values observed in the side orientation.
4. ROI specific vulnerabilities revealed that blast and resulting brain biomechanical responses affected RIOs differently. Pressure was concentrated in C, whereas equivalent stress and MPS were concentrated in CC. This emphasizes the importance of the evaluation of region specific biomechanical responses when assessing the risk of bTBI.
5. Comparison of the brain biomechanical responses between blunt impact induced mild TBI and mild bTBI simulated in this work suggested that blast and blunt impact were similar in the spatiotemporal evolution of equivalent stress and MPS. However, the spatiotemporal evolution of pressure (including peak magnitudes) was different and higher in bTBI.

## Data Availability

The original contributions presented in the study are included in the article/Supplementary Material, further inquiries can be directed to the corresponding author.
