# Supplementary material for: In silico investigation of brain tissue responses associated with mild blast traumatic brain injury
Source: Front Bioeng Biotechnol. 2026 Aug 20;14:1880112. doi: 10.3389/fbioe.2026.1880112 (PMC13538112; doi:10.3389/fbioe.2026.1880112)
Supplement: Supplementary file 1 [file Table1.docx]

Supplementary Table S. 1: 95^th^ percentile values of brain biomechanical responses for the whole brain simulated for all loading conditions.

| Equivalent charge mass (M)  (kg) | Standoff distance (R)  (m) | Peak Incident overpressure (IOP)  (kPa) | Positive phase duration (PPD)  (ms) | Positive phase impulse (PPI)  (kPa-s) | Pressure  (kPa) | Equivalent stress  (kPa) | Maximum principal strain (MPS)  (%) | Maximum principal strain rate (MPSR)  (s^-1^) | Pressure  (kPa) | Equivalent stress  (kPa) | Maximum principal strain (MPS)  (%) | Maximum principal strain rate (MPSR)  (s^-1^) |
| --- | --- | --- | --- | --- | --- | --- | --- | --- | --- | --- | --- | --- |
|  |  |  |  |  | Front Orientation | | | | Side Orientation | | | |
| 0.368 | 2.32 | 70 | 2 | 43.26 | 138.1 | 0.089 | 2.11 | 25.1 | 184.6 | 0.112 | 2.68 | 20.2 |
| 2.86 | 4.6 | 70 | 4 | 85.6 | 144.72 | 0.17 | 4 | 29.6 | 191.8 | 0.191 | 4.6 | 23.2 |
| 10.1 | 7 | 70 | 6 | 130.5 | 148.58 | 0.274 | 6.44 | 32.42 | 195.1 | 0.275 | 6.7 | 25.1 |
| 23.7 | 9.3 | 70 | 8 | 173.38 | 151.1 | 0.38 | 9 | 34.1 | 196.6 | 0.369 | 8.8 | 26.3 |
| 0.41 | 2.25 | 80 | 2 | 47.7 | 159.72 | 0.0982 | 2.34 | 28.41 | 217 | 0.126 | 3 | 23.2 |
| 3.25 | 4.5 | 80 | 4 | 94.5 | 167.2 | 0.198 | 4.59 | 33.65 | 225 | 0.22 | 5.3 | 26.4 |
| 10.82 | 6.7 | 80 | 6 | 141.5 | 172 | 0.32 | 7.5 | 39 | 230 | 0.316 | 7.6 | 28.7 |
| 26.2 | 9 | 80 | 8 | 190 | 176.55 | 0.45 | 10.6 | 39.5 | 234.1 | 0.422 | 9.95 | 30.4 |
| 0.53 | 2.3 | 90 | 2 | 54.2 | 186.26 | 0.115 | 2.71 | 32.5 | 255.2 | 0.146 | 3.5 | 26.8 |
| 3.64 | 4.4 | 90 | 4 | 103.3 | 193 | 0.227 | 5.26 | 37.85 | 262 | 0.247 | 5.9 | 30 |
| 12.27 | 6.6 | 90 | 6 | 155 | 197.1 | 0.36 | 8.5 | 41.5 | 265.4 | 0.355 | 8.5 | 32.4 |
| 29.1 | 8.8 | 90 | 8 | 206.6 | 200 | 0.51 | 11.9 | 44.1 | 267.8 | 0.477 | 11.1 | 34.1 |
| 0.53 | 2.2 | 100 | 2 | 56.8 | 207 | 0.12 | 2.88 | 35.2 | 285.2 | 0.155 | 3.7 | 29.4 |
| 3.95 | 4.3 | 100 | 4 | 110.86 | 217.71 | 0.253 | 6 | 42 | 298.2 | 0.274 | 6.6 | 33.6 |
| 13.65 | 6.5 | 100 | 6 | 167.6 | 223.26 | 0.412 | 9.64 | 46.2 | 300.1 | 0.391 | 9.3 | 35.1 |
| 31.6 | 8.6 | 100 | 8 | 221.7 | 226.4 | 0.58 | 13.5 | 48.9 | 305.8 | 0.53 | 12.2 | 38.2 |
| 0.607 | 2.2 | 110 | 2 | 61.8 | 232.26 | 0.135 | 3.2 | 38.7 | 321.4 | 0.17 | 4.1 | 32.7 |
| 4.53 | 4.3 | 110 | 4 | 120.7 | 244.57 | 0.294 | 6.86 | 46.3 | 336.6 | 0.304 | 7.3 | 37.5 |
| 14.93 | 6.4 | 110 | 6 | 180 | 251 | 0.462 | 10.8 | 50.83 | 341.7 | 0.44 | 10.4 | 40.2 |
| 35 | 8.5 | 110 | 8 | 238.5 | 255.28 | 0.65 | 15 | 53.7 | 344.8 | 0.594 | 13.5 | 42.4 |
| 0.686 | 2.2 | 120 | 2 | 66.6 | 258.17 | 0.149 | 3.5 | 42.33 | 358.6 | 0.186 | 4.4 | 36.1 |
| 5.12 | 4.3 | 120 | 4 | 130.2 | 273.35 | 0.322 | 7.5 | 50.37 | 375.8 | 0.338 | 8.1 | 41.2 |
| 16.9 | 6.4 | 120 | 6 | 194 | 280 | 0.512 | 11.8 | 55.4 | 382.2 | 0.5 | 11.6 | 44.4 |
| 39.55 | 8.5 | 120 | 8 | 257.3 | 283.62 | 0.728 | 16.7 | 58.56 | 398.8 | 0.663 | 15.2 | 47.8 |
| 0.767 | 2.2 | 130 | 2 | 71.4 | 285.86 | 0.164 | 3.86 | 46 | 395.1 | 0.194 | 4.58 | 39.2 |
| 5.73 | 4.3 | 130 | 4 | 140 | 300.75 | 0.355 | 8.2 | 54.7 | 416 | 0.373 | 8.9 | 45.3 |
| 18.9 | 6.4 | 130 | 6 | 208 | 309.62 | 0.561 | 13 | 60 | 423.8 | 0.591 | 12.1 | 48.6 |
| 44.2 | 8.5 | 130 | 8 | 275.7 | 313.2 | 0.827 | 18.8 | 63.36 | 427 | 0.81 | 15.8 | 50.6 |
| 0.85 | 2.2 | 140 | 2 | 76.1 | 312.3 | 0.18 | 4.2 | 49.67 | 436.1 | 0.222 | 5.3 | 43.1 |
| 6.35 | 4.3 | 140 | 4 | 148.8 | 330 | 0.384 | 8.8 | 59.1 | 457.4 | 0.41 | 9.5 | 50 |
| 21.93 | 6.5 | 140 | 6 | 225 | 339.1 | 0.631 | 14.5 | 64.78 | 466.3 | 0.626 | 14.1 | 52.8 |
| 50.8 | 8.6 | 140 | 8 | 298 | 343.84 | 0.923 | 21 | 64.5 | 471 | 0.843 | 18.2 | 55.5 |
| 0.893 | 2.2 | 145 | 2 | 78.5 | 327 | 0.19 | 4.45 | 51.5 | 455.8 | 0.225 | 5.3 | 44.9 |
| 6.67 | 4.3 | 145 | 4 | 154 | 344.74 | 0.402 | 9.24 | 61.2 | 479.3 | 0.425 | 10 | 51.4 |
| 23 | 6.5 | 145 | 6 | 232 | 354.5 | 0.662 | 15.2 | 67.1 | 487.6 | 0.645 | 14.5 | 55 |
| 53.31 | 8.6 | 145 | 8 | 307 | 359.2 | 0.964 | 21.6 | 71 | 495.7 | 0.88 | 18.9 | 57.5 |
