# Supplementary material for: In silico investigation of brain tissue responses associated with mild blast traumatic brain injury
Source: Front Bioeng Biotechnol. 2026 Aug 20;14:1880112. doi: 10.3389/fbioe.2026.1880112 (PMC13538112; doi:10.3389/fbioe.2026.1880112)
Supplement: Supplementary file 2 [file Table2.docx]

Supplementary Table S. 2: Median and interquartile range (25^th^-75^th^) of biomechanical responses for the whole brain and ROIs under head impact using Worcester Head Injury Model (WHIM) [137].

| Biomechanical responses  Median and (25^th^-75^th^) | Whole brain | Cerebrum  (C) | Cerebellum (CB) | Corpus callosum  (CC) | Brainstem (BS) |
| --- | --- | --- | --- | --- | --- |
| Coup pressure (kPa) | 67.12  (52.41 to 79.30) | 69.69  (48.27 to 81.48) | 17.51  (4.7 to 36.85) | 28.53  (21.38 to 40.99) | 11.58  (3.20 to 31.56) |
| Contrecoup pressure  (kPa) | -58.27  (-76.14 to 45.62) | -48.61  (-75.19 to 35.85) | -47.57  (-63.37 to 28.93) | -25.97  (-36.82 to 12.53) | -50.12  (-59.47 to 28.32) |
| Equivalent stress (kPa) | 1.59  (0.98 to 2.45) | 1.79  (1.06 to 2.56) | 0.59  (0.35 to 0.98) | 1.15  (0.67 to 1.66) | 1.25  (0.77 to 3.11) |
| Maximum principal strain (MPS) (%) | 16  (13 to 20) | 18  (14 to 21) | 9  (6 to 11) | 13  (9 to 16) | 14  (12 to 22) |
| Maximum principal strain rate (MPSR)  (s^-1^) | 28.82  (19.76 to 37.17) | 30.53  (20.48 to 37.33) | 19.23  (13.94 to 26.72) | 25.68  (16.51 to 33.65) | 30.18  (24.21 to 49.56) |
